# Supplementary material for: Dietary Inflammatory Index and Its Association with the Prevalence of Coronary Heart Disease among 45,306 US Adults
Source: Nutrients. 2022 Oct 28;14(21):4553. doi: 10.3390/nu14214553 (PMC9656485; doi:10.3390/nu14214553)
Supplement: Supplementary file 1 [file nutrients-14-04553-s001.zip › Table_S1.pdf]

Table S1. Baseline Characteristics of All Participants Grouped by Sex.

Variables	Male				Female				*P* value between male and female
	**Overall (*n* = 22517)**	**Non-CHD (*n* = 21654)**	**CHD (*n* = 863)**	***P* value**	**Overall (*n* = 22507)**	**Non-CHD (*n* = 22115)**	**CHD (*n* = 392)**	***P* value**	
Age, years	47.00 [33.00, 62.00]	46.00 [33.00, 61.00]	67.00 [60.00, 73.00]	<0.001***	46.00 [32.00, 61.00]	45.00 [32.00, 60.00]	66.00 [59.00, 73.00]	<0.001***	<0.001***
Race, *n* (%)									<0.001***
Non-Hispanic White	9488 (43.4)	8830 (42.5)	658 (61.2)	<0.001***	9834 (41.9)	9570 (41.7)	264 (52.8)	<0.001***	
Non-Hispanic Black	4713 (21.6)	4558 (21.9)	155 (14.4)		5108 (21.8)	5020 (21.9)	88 (17.6)		
Mexican American	3969 (18.2)	3838 (18.5)	131 (12.2)		4341 (18.5)	4270 (18.6)	71 (14.2)		
Other Hispanic	1720 (7.9)	1657 (8.0)	63 (5.9)		2098 (8.9)	2062 (9.0)	36 (7.2)		
Other	1967 (9.0)	1899 (9.1)	68 (6.3)		2068 (8.8)	2027 (8.8)	41 (8.2)		<0.001***
Smoking, *n* (%)	5698 (26.1)	5494 (26.5)	204 (19.0)	<0.001***	4293 (18.3)	4181 (18.2)	112 (22.4)	0.02*	<0.001***
Drinking, *n* (%)	15437 (75.4)	14825 (76.1)	612 (61.9)		13581 (63.3)	13389 (63.7)	192 (42.5)	<0.001***	<0.001***
Education level, *n* (%)								<0.001***	<0.001***
Below high school	2584 (11.8)	2416 (11.6)	168 (15.6)	<0.001***	2485 (10.6)	2391 (10.4)	94 (18.8)		
High school	8623 (39.5)	8216 (39.6)	407 (37.9)		8614 (36.8)	8378 (36.5)	236 (47.2)		
Above high school	10635 (48.7)	10135 (48.8)	500 (46.5)		12329 (52.6)	12159 (53.0)	170 (34.0)		<0.001***
BMI, kg/m^2	27.80 [24.60, 31.52]	27.70 [24.51, 31.40]	29.20 [26.14, 33.32]	<0.001***	28.38 [24.12, 33.69]	28.34 [24.10, 33.65]	29.51 [25.31, 34.77]	<0.001***	<0.001***
SBP, mmHg	123.00 [114.00, 134.00]	122.00 [114.00, 133.00]	127.00 [116.00, 140.00]	<0.001***	117.00 [107.00, 131.00]	117.00 [107.00, 131.00]	131.00 [118.00, 147.00]	<0.001***	<0.001***
DBP, mmHg	73.00 [65.00, 80.00]	73.00 [65.00, 80.00]	69.00 [61.00, 77.00]	<0.001***	70.00 [62.00, 77.00]	70.00 [62.00, 77.00]	66.00 [58.00, 74.00]	<0.001***	<0.001***
HBP, *n* (%)	12762 (58.4)	12535 (60.3)	227 (21.1)	<0.001***	14289 (60.9)	14212 (61.9)	77 (15.4)	<0.001***	<0.001***
Income > 20,000USD	15746 (75.5)	15003 (75.7)	743 (71.4)	0.002**	16230 (72.4)	15972 (72.7)	258 (54.8)	<0.001***	<0.001***
FBG, mmol/L	5.63 [5.24, 6.22]	5.61 [5.22, 6.16]	6.16 [5.55, 7.22]	<0.001***	5.38 [4.96, 5.88]	5.36 [4.94, 5.88]	6.00 [5.33, 7.65]	<0.001***	<0.001***
FBI, mmol/L	58.98 [37.32, 97.54]	58.26 [36.96, 96.42]	72.90 [47.10, 122.13]	<0.001***	59.34 [38.16, 95.64]	59.10 [38.10, 95.43]	70.71 [42.76, 107.50]	0.002**	0.948
HbA1c, %	5.50 [5.20, 5.80]	5.50 [5.20, 5.80]	5.80 [5.50, 6.60]	<0.001***	5.40 [5.10, 5.80]	5.40 [5.10, 5.80]	5.80 [5.50, 6.80]	<0.001***	<0.001***
TG, mmol/L	1.29 [0.89, 1.93]	1.28 [0.88, 1.92]	1.45 [0.99, 2.10]	<0.001***	1.16 [0.80, 1.73]	1.15 [0.79, 1.72]	1.46 [1.02, 2.34]	<0.001***	<0.001***
TC, mmol/L	4.94 [4.24, 5.66]	4.97 [4.29, 5.69]	4.34 [3.72, 5.12]	<0.001***	5.04 [4.40, 5.79]	5.07 [4.40, 5.79]	4.78 [4.16, 5.65]	<0.001***	<0.001***
HDL-C, mmol/L	1.16 [0.98, 1.42]	1.17 [0.98, 1.42]	1.11 [0.93, 1.31]	<0.001***	1.42 [1.19, 1.73]	1.42 [1.19, 1.73]	1.29 [1.09, 1.58]	<0.001***	<0.001***
LDL-C, mmol/L	2.97 [2.35, 3.60]	3.00 [2.40, 3.62]	2.38 [1.81, 2.95]	<0.001***	2.90 [2.35, 3.54]	2.90 [2.35, 3.54]	2.69 [2.00, 3.35]	0.007**	<0.001***
Alt, u	25.00 [19.00, 34.00]	25.00 [19.00, 34.00]	23.00 [18.00, 29.00]	<0.001***	18.00 [14.00, 23.00]	18.00 [14.00, 23.00]	19.00 [15.00, 23.00]	0.444	<0.001***
Ast, u	24.00 [21.00, 29.00]	24.00 [21.00, 29.00]	24.00 [20.00, 29.00]	0.036*	21.00 [18.00, 25.00]	21.00 [18.00, 25.00]	22.00 [19.00, 26.00]	<0.001***	<0.001***
RBC, ×10^9/L	4.97 [4.68, 5.24]	4.97 [4.69, 5.25]	4.76 [4.41, 5.06]	<0.001***	4.44 [4.18, 4.70]	4.44 [4.18, 4.70]	4.40 [4.09, 4.69]	<0.001***	<0.001***
WBC, ×10^9/L	6.90 [5.70, 8.30]	6.90 [5.60, 8.20]	7.10 [5.90, 8.40]	0.002**	7.10 [5.80, 8.60]	7.10 [5.80, 8.60]	7.20 [6.00, 8.90]	0.145	<0.001***
PLT, ×10^6/L	233.00 [198.00, 272.00]	234.00 [200.00, 273.00]	206.00 [173.00, 247.00]	<0.001***	261.00 [222.00, 308.00]	261.00 [222.00, 308.00]	247.00 [203.00, 292.00]	<0.001***	<0.001***
Monocyte, ×10^9/L	0.60 [0.40, 0.70]	0.60 [0.40, 0.70]	0.60 [0.50, 0.70]	<0.001***	0.50 [0.40, 0.60]	0.50 [0.40, 0.60]	0.50 [0.50, 0.70]	<0.001***	<0.001***
LY, ×10^9/L	2.00 [1.60, 2.50]	2.00 [1.60, 2.50]	1.80 [1.40, 2.30]	<0.001***	2.10 [1.70, 2.60]	2.10 [1.70, 2.60]	2.10 [1.60, 2.52]	0.042*	<0.001***
NE, ×10^9/L	3.90 [3.10, 5.00]	3.90 [3.10, 5.00]	4.20 [3.30, 5.40]	<0.001***	4.10 [3.10, 5.30]	4.10 [3.10, 5.30]	4.30 [3.20, 5.60]	0.065	<0.001***
Hemoglobin, g/L	15.10 [14.40, 15.90]	15.10 [14.40, 15.90]	14.60 [13.70, 15.50]	<0.001***	13.40 [12.60, 14.10]	13.40 [12.60, 14.10]	13.30 [12.50, 14.10]	0.499	<0.001***
eGFR, ml/min/1.73m^2	95.17 [80.64, 109.00]	96.12 [81.79, 109.63]	75.71 [60.63, 88.34]	<0.001***	99.96 [83.60, 116.00]	100.47 [84.26, 116.33]	75.87 [57.24, 92.13]	<0.001***	<0.001***
DII	1.29 [-0.19, 2.59]	1.27 [-0.20, 2.59]	1.61 [0.09, 2.80]	<0.001***	2.12 [0.69, 3.19]	2.11 [0.68, 3.18]	2.58 [1.33, 3.48]	<0.001***	<0.001***
DM, *n* (%)	3811 (17.4)	3338 (16.1)	473 (44.0)	<0.001***	3592 (16.3)	3368 (15.6)	224 (45.0)	<0.001***	<0.001***
Angina, *n* (%)	613 (2.8)	251 (1.2)	362 (34.3)	<0.001***	478 (2.0)	326 (1.4)	152 (31.3)	<0.001***	<0.001***
Heart attack, *n* (%)	1130 (5.2)	534 (2.6)	596 (55.8)	<0.001***	552 (2.4)	329 (1.4)	223 (45.0)	<0.001***	<0.001***
HF, *n* (%)	697 (3.2)	349 (1.7)	348 (33.0)	<0.001***	492 (2.1)	344 (1.5)	148 (30.1)	<0.001***	<0.001***
Stroke, *n* (%)	735 (3.4)	573 (2.8)	162 (15.1)	<0.001***	727 (3.1)	639 (2.8)	88 (17.7)	<0.001***	0.119

Variables are presented as the mean ± standard deviation (SD) (normal distribution), the median (interquartile range) (skewed distribution) or number with percent (categorical). SD, standard deviation; BMI, body mass index; SBP, systolic blood pressure; DBP, diastolic blood pressure; HBP, hypertension; FBG, fasting blood glucose; FBI, fasting blood insulin; HbA1c, glycated hemoglobin; TG, triglycerides; TC, total cholesterol; HDL-C, high-density lipoprotein cholesterol; LDL-C, low-density lipoprotein cholesterol; Alt, alanine transaminase; Ast, glutamic oxalic

transaminase; RBC, red blood cells; WBC, white blood cells; PLT, platelets; LY, lymphocytes; NE, neutrophils; eGFR, estimated glomerular filtration rate; DII, dietary inflammatory index; DM, diabetes; HF, heart failure. *** P value<0.001, ** P value<0.01, * P value<0.05.
