# Supplementary material for: Dietary Inflammatory Index and Its Association with the Prevalence of Coronary Heart Disease among 45,306 US Adults
Source: Nutrients. 2022 Oct 28;14(21):4553. doi: 10.3390/nu14214553 (PMC9656485; doi:10.3390/nu14214553)
Supplement: Supplementary file 1 [file nutrients-14-04553-s001.zip › Table_S2.pdf]

Table S2. Comparison of each Component of DII Scores of All Participants Grouped by Sex.

**Variables**	**Male**				**Female**				***P* value between male and female**
	**Overall (*n* = 22517)**	**Non−CHD (*n* = 21654)**	**CHD (*n* = 863)**	***P* value**	**Overall (*n* = 22507)**	**Non−CHD (*n* = 22115)**	**CHD (*n* = 392)**	***P* value**	
DII	1.29 [−0.18, 2.60]	1.28 [−0.19, 2.59]	1.60 [0.03, 2.79]	0.001**	2.17 [0.76, 3.23]	2.17 [0.75, 3.22]	2.48 [1.23, 3.46]	<0.001***	<0.001***
Energy	0.10 [−0.13, 0.18]	0.11 [−0.12, 0.18]	−0.07 [−0.17, 0.13]	<0.001***	−0.12 [−0.18, 0.07]	−0.12 [−0.18, 0.08]	−0.17 [−0.18, −0.08]	<0.001***	<0.001***
Protein	0.00 [−0.09, 0.09]	0.00 [−0.09, 0.10]	−0.07 [−0.10, 0.04]	<0.001***	−0.08 [−0.10, 0.02]	−0.08 [−0.10, 0.02]	−0.09 [−0.10, −0.05]	<0.001***	<0.001***
Carbohydrate	0.01 [−0.02, 0.02]	0.01 [−0.02, 0.02]	−0.01 [−0.02, 0.02]	<0.001***	−0.02 [−0.02, 0.01]	−0.02 [−0.02, 0.01]	−0.02 [−0.02, −0.01]	<0.001***	<0.001***
Dietary fiber	0.14 [−0.17, 0.29]	0.15 [−0.16, 0.29]	−0.03 [−0.23, 0.23]	<0.001***	−0.10 [−0.26, 0.19]	−0.10 [−0.26, 0.19]	−0.21 [−0.28, 0.02]	<0.001***	<0.001***
Total fatty acid	0.28 [0.00, 0.28]	0.28 [−0.05, 0.28]	0.28 [0.28, 0.28]	<0.001***	0.28 [0.28, 0.28]	0.28 [0.28, 0.28]	0.28 [0.28, 0.28]	<0.001***	<0.001***
Total saturated fatty acid	0.31 [−0.44, 0.61]	0.30 [−0.45, 0.61]	0.36 [−0.31, 0.63]	0.001**	0.50 [−0.03, 0.64]	0.50 [−0.04, 0.64]	0.57 [0.20, 0.65]	<0.001***	<0.001***
Total monounsaturated fatty acid	−0.01 [−0.11, 0.11]	−0.01 [−0.11, 0.11]	−0.08 [−0.11, 0.11]	<0.001***	−0.10 [−0.11, 0.07]	−0.10 [−0.11, 0.08]	−0.11 [−0.11, 0.01]	<0.001***	<0.001***
Total polyunsaturated fatty acid	−0.08 [−0.32, 0.30]	−0.07 [−0.32, 0.31]	−0.24 [−0.35, 0.09]	<0.001***	−0.27 [−0.36, 0.02]	−0.27 [−0.35, 0.02]	−0.33 [−0.36, −0.17]	<0.001***	<0.001***
n3 polyunsaturated fatty acid	0.00 [−0.01, 0.01]	0.00 [−0.01, 0.01]	0.00 [−0.01, 0.01]	<0.001******	0.01 [−0.01, 0.01]	0.00 [−0.01, 0.01]	0.01 [0.00, 0.01]	<0.001***	<0.001***
n6 polyunsaturated fatty acid	−0.22 [−0.34, 0.18]	−0.23 [−0.34, 0.18]	−0.04 [−0.32, 0.25]	<0.001***	0.02 [−0.31, 0.29]	0.02 [−0.31, 0.28]	0.18 [−0.18, 0.31]	<0.001***	<0.001***
Cholesterol	0.00 [−0.15, 0.12]	0.00 [−0.15, 0.12]	0.06 [−0.08, 0.15]	<0.001	0.12 [0.01, 0.18]	0.11 [0.01, 0.18]	0.15 [0.06, 0.20]	<0.001***	<0.001***
Vitamin A	0.26 [0.09, 0.34]	0.26 [0.09, 0.34]	0.27 [0.12, 0.33]	0.827	0.28 [0.14, 0.34]	0.28 [0.14, 0.34]	0.30 [0.19, 0.34]	<0.001***	<0.001***
Vitamin B1	0.01 [−0.06, 0.06]	0.01 [−0.06, 0.06]	0.02 [−0.04, 0.07]	<0.001***	0.05 [0.00, 0.08]	0.05 [0.00, 0.08]	0.06 [0.02, 0.08]	<0.001***	<0.001***
Vitamin B2	−0.02 [−0.06, 0.02]	−0.03 [−0.06, 0.02]	−0.02 [−0.05, 0.02]	<0.001***	0.01 [−0.03, 0.04]	0.01 [−0.04, 0.04]	0.01 [−0.02, 0.04]	<0.001***	<0.001***
Vitamin B6	−0.19 [−0.34, 0.05]	−0.19 [−0.34, 0.04]	−0.11 [−0.30, 0.11]	<0.001***	0.01 [−0.22, 0.18]	0.00 [−0.22, 0.18]	0.08 [−0.12, 0.22]	<0.001***	<0.001***
Vitamin B12	−0.02 [−0.07, 0.06]	−0.02 [−0.07, 0.06]	−0.04 [−0.08, 0.04]	<0.001***	−0.06 [−0.08, 0.00]	−0.06 [−0.08, 0.00]	−0.07 [−0.09, −0.02]	<0.001***	<0.001***
Vitamin C	0.36 [−0.08, 0.41]	0.36 [−0.08, 0.41]	0.36 [0.00, 0.41]	0.537	0.37 [0.02, 0.41]	0.37 [0.02, 0.41]	0.38 [0.13, 0.41]	<0.001***	<0.001***
Vitamin D	0.44 [0.24, 35.90]	0.44 [0.24, 35.70]	0.44 [0.27, 40.70]	0.277	0.44 [0.34, 37.85]	0.44 [0.34, 37.75]	0.44 [0.38, 43.30]	0.199	<0.001***
Vitamin E	0.30 [−0.36, 0.42]	0.29 [−0.36, 0.42]	0.39 [−0.23, 0.42]	<0.001***	0.40 [−0.03, 0.42]	0.39 [−0.03, 0.42]	0.41 [0.25, 0.42]	<0.001***	<0.001***
Folate	0.01 [−0.02, 0.03]	0.01 [−0.02, 0.03]	0.00 [−0.02, 0.03]	<0.001***	−0.01 [−0.03, 0.02]	−0.01 [−0.03, 0.02]	−0.02 [−0.03, 0.01]	<0.001***	<0.001***
β−carotene	0.03 [−0.25, 0.24]	0.02 [−0.25, 0.24]	0.10 [−0.15, 0.29]	<0.001***	0.20 [−0.02, 0.33]	0.19 [−0.02, 0.33]	0.26 [0.07, 0.37]	<0.001***	<0.001***
Niacin	−0.18 [−0.31, 0.20]	−0.18 [−0.31, 0.19]	−0.03 [−0.30, 0.26]	<0.001***	0.15 [−0.21, 0.29]	0.15 [−0.21, 0.29]	0.23 [−0.04, 0.30]	<0.001***	<0.001***
Iron	−0.18 [−0.19, −0.09]	−0.18 [−0.19, −0.09]	−0.16 [−0.19, −0.02]	<0.001***	−0.11 [−0.18, 0.04]	−0.11 [−0.18, 0.04]	−0.04 [−0.16, 0.09]	<0.001***	<0.001***
Magnesium	0.18 [0.05, 0.19]	0.18 [0.05, 0.19]	0.18 [0.11, 0.19]	<0.001***	0.19 [0.14, 0.19]	0.19 [0.14, 0.19]	0.19 [0.16, 0.19]	0.012*	<0.001***
Zinc	0.54 [0.40, 0.56]	0.54 [0.40, 0.56]	0.54 [0.38, 0.56]	0.721	0.54 [0.36, 0.56]	0.54 [0.36, 0.56]	0.55 [0.46, 0.56]	0.001***	0.434
Selenium	0.08 [0.08, 0.08]	0.08 [0.08, 0.08]	0.08 [0.08, 0.08]	<0.001***	0.08 [0.08, 0.08]	0.08 [0.08, 0.08]	0.08 [0.08, 0.08]	0.096	<0.001***

Variables are presented as the mean ± standard deviation (SD) (normal distribution), the median (interquartile range) (skewed distribution). DII, dietary inflammatory index; CHD, coronary heart disease; MUFA, monounsaturated fatty acids; PUFA, polyunsaturated fatty acids. *** *P* value<0.001, ** *P* value<0.01, * *P* value<0.05.
